# Pathophysiology and mechanisms of hearing impairment related to neonatal infection diseases

**DOI:** 10.3389/fmicb.2023.1162554

**Published:** 2023-04-14

**Authors:** Daniela Capra, Marcos F. DosSantos, Carolina K. Sanz, Lionete Gall Acosta Filha, Priscila Nunes, Manoela Heringer, Adriana Ximenes-da-Silva, Luciana Pessoa, Juliana de Mattos Coelho-Aguiar, Anna Carolina Carvalho da Fonseca, Carmelita Bastos Mendes, Lanni Sarmento da Rocha, Sylvie Devalle, Paulo Niemeyer Soares Filho, Vivaldo Moura-Neto

**Affiliations:** ^1^Laboratório de Morfogênese Celular (LMC), Instituto de Ciências Biomédicas (ICB), Universidade Federal do Rio de Janeiro (UFRJ), Rio de Janeiro, Brazil; ^2^Laboratório de Biomedicina do Cérebro, Instituto Estadual do Cérebro Paulo Niemeyer (IECPN), Secretaria de Estado de Saúde, Rio de Janeiro, Brazil; ^3^Programa de Pós-Graduação em Neurociência Translacional, Instituto Nacional de Neurociência Translacional (INNT-UFRJ), Rio de Janeiro, Rio de Janeiro, Brazil; ^4^Programa de Pós-Graduação em Odontologia (PPGO), Universidade Federal do Rio de Janeiro (UFRJ), Rio de Janeiro, Brazil; ^5^Laboratório de Propriedades Mecânicas e Biologia Celular (PropBio), Departamento de Prótese e Materiais Dentários, Faculdade de Odontologia, Universidade Federal do Rio de Janeiro (UFRJ), Rio de Janeiro, Brazil; ^6^Setor de Fisiologia, Universidade Federal de Alagoas (UFAL), Alagoas, Brazil; ^7^Programa de Pós-Graduação em Anatomia Patológica, Hospital Universitário Clementino Fraga Filho (HUCFF), Universidade Federal do Rio de Janeiro, Rio de Janeiro, Brazil

**Keywords:** hearing loss, congenital Zika virus, toxoplasmosis, cytomegalovirus, HIV, Rubeola, COVID-19, human induced pluripotent stem cells

## Abstract

The inner ear, the organ of equilibrium and hearing, has an extraordinarily complex and intricate arrangement. It contains highly specialized structures meticulously tailored to permit auditory processing. However, hearing also relies on both peripheral and central pathways responsible for the neuronal transmission of auditory information from the cochlea to the corresponding cortical regions. Understanding the anatomy and physiology of all components forming the auditory system is key to better comprehending the pathophysiology of each disease that causes hearing impairment. In this narrative review, the authors focus on the pathophysiology as well as on cellular and molecular mechanisms that lead to hearing loss in different neonatal infectious diseases. To accomplish this objective, the morphology and function of the main structures responsible for auditory processing and the immune response leading to hearing loss were explored. Altogether, this information permits the proper understanding of each infectious disease discussed.

**GRAPHICAL ABSTRACT:**
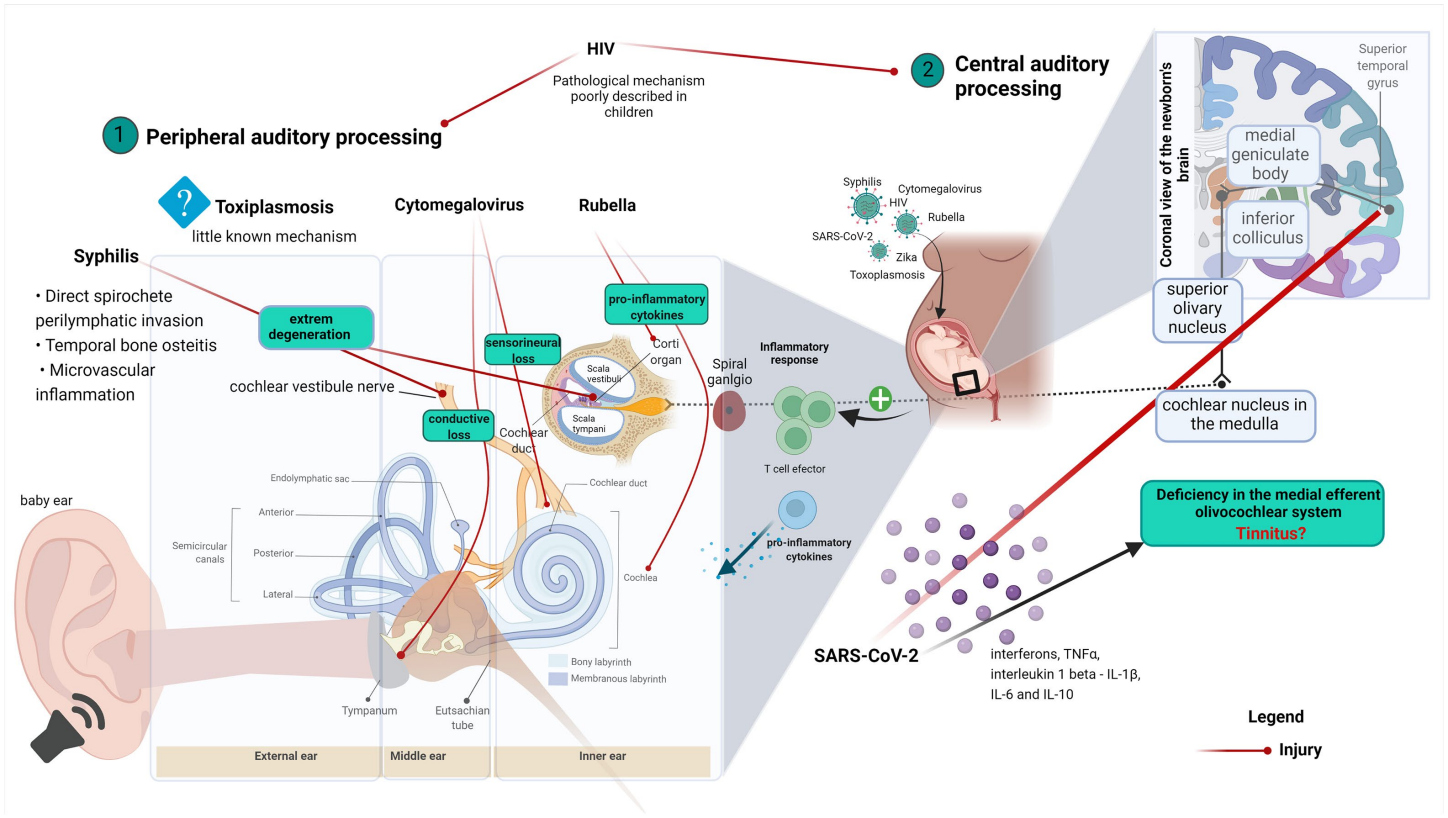
Graphical abstract explaining the correlation between ear anatomy, hearing loss, and virus infection diseases. (1) Virus effects on the peripheral auditory process and (2) Virus effects on the central auditory process.

## 1. Introduction

The human cochlea is a shell-shaped rigid labyrinth that transforms acoustic stimuli into electrical impulses. However, its normal functioning may be altered in several circumstances, mostly due to interruptions in the auditory pathway through its course from the cochlea to the brainstem (Nadol, 1988).

Pregnancy is a process that changes immune system, making pregnant women susceptible to infections (Vale et al., 2021). Endemic infectious diseases that arise in pregnant women can compromise maternal and fetal health (Hotez et al., 2007; Ficenec et al., 2019). For instance, many infectious diseases result in sensorineural hearing loss caused either by a direct cytotoxic effect related to the activity of the pathogenic agent or by indirect tissue damage related to an inflammatory response that occurs in the cochlea and associated structures (Abbas and Rivolta, 2019).

Advances in public health have led to a significant reduction in the incidence of post-infection deafness, particularly in developed countries. For example, measles, mumps, and rubella vaccination campaigns have almost eliminated the deafness caused by these viruses. In some cases, antiviral therapies are also adopted to treat infectious diseases that affect the auditory system; an example is the use of ganciclovir. This drug halts the progression of congenital cytomegalovirus (CMV) syndrome (Abbas and Rivolta, 2019). Despite significant advances in prevention and treatment, the World Health Organization (WHO) estimates that almost 60% of hearing loss in childhood is still associated with ear infections and complications in childbirth, which can be avoided by adopting appropriate public health measures (WHO, 2021).

In this paper, the authors review the main anatomical and physiological aspects of the auditory pathway and discuss the pathophysiological effects and the immune response of infectious diseases related to hearing impairment.

## 2. General organization of the cochlea

The cochlea represents the peripheral organ of hearing that converts sound energy into electrical signals, which can be interpreted by the brain. The human cochlea is a seashell-shaped part of a bony labyrinth, located within the petrous part of the temporal bone (Nadol, 1988). In mammals, the cochlea is a coiled fluid-filled tube composed of three chambers: the vestibular ramp (*scala vestibuli*), the tympanic ramp (*scala tympani*), and the cochlear duct (*scala media*; Cormack et al., 2015).

The *scala media* (membranous labyrinth) contains the endolymph, a liquid with an ionic composition comparable to the intracellular fluid, with high concentrations of K^+^. Well-developed tight junctions prevent the fluid from leaking into the intercellular space. The endolymph flows into the endolymphatic sac, *via* the endolymphatic duct. On the other hand, the perilymph, which contains high concentrations of Na^+^ similar to extracellular fluids (Raphael and Altschuler, 2003), fills up the vestibular ramp (*scala vestibuli*) and the tympanic ramp (*scala tympani*).

The sensory cells of the cochlea are called hair cells. These are highly specialized mechanosensors residing in the organ of the Corti, which is located inside the cochlear duct. Two types of hair cells are classified into inner hair cells (IHC) and outer hair cells (OHC). IHCs are considered the true sensory cell type. They propagate impulses *via* the auditory nerve. Noteworthy, significant differences exist in the innervation pattern of each IHC subgroup. These differences are related to the number of nerve terminals, their degree of branching, and the number of synapses per afferent terminal. The morphology of the synapses between the afferent fibers and the IHCs is remarkably preserved across species. Conversely, efferent terminals do not end directly on IHCs. In fact, they end in the dendritic terminals near the base of IHCs (Nadol, 1988). On the other hand, OHCs act as mechanical amplifiers, enhancing weak sounds in the cochlea. They are regulated by efferent inputs that arise from the brainstem (Raphael and Altschuler, 2003; Takahashi et al., 2016; Harris et al., 2017).

The Organ of Corti is a specialized structure that transforms mechanical vibrations into nerve impulses. This vibration occurs according to the frequency of the sound waves that reach the inner ear. This is one of the mechanisms responsible for the selectivity of the cochlear frequency (Von Békésy, 1960).

The modiolus is the osseous cone-shaped central pillar of the cochlea from which the osseous spiral lamina projects into the bony canal of the cochlea, resulting in a structure with a screw-like appearance. The osseous cochlear canal spirals for about 2.5–2.75 turns around its central bony core, the modiolus, and is approximately 35 mm in length (Wang et al., 2016).

## 3. Spiral ganglion and neural fibers (afferent neurons)

The auditory nerve is the sole supply route of the auditory information from hair cells to the brain. It carries out information related to sound frequency, intensity, timbre, and pitch (Petitpré et al., 2018). The mammalian cochlea is innervated by two types of sensory neurons, classified as type I and type II sensory neurons or spiral ganglion neurons (SGNs). Type I and type II SGNs convey auditory information from the cochlear sensory receptors to the central nervous system (CNS; Reid et al., 2004). The central afferents of these SGN neurons converge to form the auditory nerve, which connects to the cochlear nuclei in the brainstem. Most of the SGNs are classified as type I neurons, these are myelinated fibers that contact the IHCs and are the main pathway of auditory signals. Moreover, three subtypes of type I SGNs have been identified in the adult cochlea: Ia neurons have been characterized by the expression of Calb1, Pou4f1, Runx1, and calretinin (CR); Ib neurons have been distinguished by the expression Lypd1, Runx1, and Pou4f1, while Ic neurons, have been marked by the expression of Rxrg, Pcdh20, and CR (Petitpré et al., 2018). Using different combinations of specific markers, the proportion of the four types of SGNs was found to be relatively constant throughout the length of the cochlea. In this regard, 7% of SGNs have been classified as type II neurons, 26% as type Ia neurons, 24% as type Ib neurons, and 43% as type Ic neurons (Petitpré et al., 2018). Remarkably, synaptic mechanisms differ substantially between type I and type II SGNs. Type I SGNs contact single IHCs and are associated with the acoustic analysis, whereas type II SGNs neurons arborize considerably when contacting the supporting cells and the OHCs. In fact, type II SGNs serve as cochlear afferents that can also be modulated by ATP (Weisz et al., 2009).

Noteworthy, emerging evidence suggests that aquaporins (AQP), particularly aquaporin-4 (AQP4), play an important role in regulating inner ear fluid homeostasis and, consequently, contribute to the maintenance of the ionic gradient required for hearing and balancing sensory excitability (Beitz et al., 1999; Mhatre et al., 2002).

## 4. Immune response to hearing loss

The presence of resident macrophages/microglia in the inner ear has been extensively described in animal models, especially through the study of their modulation in the context of injury (Bhave et al., 1998; Torres et al., 1999; Fuentes-Santamaría et al., 2012; Janz and Illing, 2014). However, only recently the identification of the macrophages/microglia markers CD163+, Iba1+, and CD68+ has been demonstrated in the human inner ear (O’malley et al., 2015). This study presents immunostaining with neuron and glial markers within the cochlea.

It has been suggested that the increase of cytokines and chemokines synthesis by diverse types of inner ear cells is related to the presence of immune cells. This process is associated with cochlear damage and hearing loss (Satoh et al., 2003; Fujioka et al., 2006). Wakabayashi and colleagues observed a decreased migration of Iba-1 cochlear macrophages after using the anti-IL-6 receptor antibody MR16-1. Moreover, the same study showed that the use of the anti-IL-6 treatment was able to increase the auditory brainstem response (ABR) in an experimental model of noise-induced cochlear damage (Wakabayashi et al., 2010).

Regarding the participation of macrophages/microglia in auditory impairment, evidence regarding the role of macrophages/microglia in hearing loss caused by infectious diseases is still lacking. A few papers explored this topic. Schachtele and colleagues reported the death of hair cells 21 days after cytomegalovirus infection. Moreover, the author showed the production of reactive oxygen species produced by the cochlea-infiltrated macrophages until 14 days after the initial infection (Schachtele et al., 2011). The primary role of macrophages as cells of the innate immune defense is well established, and production of reactive oxygen species constitutes one of the main mechanisms for eliminating invading pathogens (Forman and Torres, 2002; Paiva and Bozza, 2014). Taken together, these findings suggest that the activation of macrophages/microglia takes place following an ear infection. Nonetheless, to what extent this process is associated with hearing loss is still to be determined. This narrative review aims to summarize the available evidence and clarify possible contribution virus infections to hearing loss.

## 5. Neonatal infections in the context of hearing loss

### 5.1. Toxoplasmosis

Toxoplasmosis is an infection caused by the protozoan *Toxoplasma gondii*, an obligate intracellular parasite, which is acquired by eating contaminated food or water. During pregnancy, transplacental transmission from the mother to the fetus may occur, and when occurring in early pregnancy, inflammation and necrosis can be observed, particularly in the fetal CNS (Dubey et al., 2005; Salviz et al., 2013). Both inflammation and replication of parasites may destroy the white matter and block the aqueduct of *Sylvius* of the fetal brain (Robert-Gangneux and Dardé, 2012).

Most newborns with congenital toxoplasmosis have subclinical infections at birth (Montoya and Liesenfeld, 2004). Among the children diagnosed with congenital toxoplasmosis, several die shortly after birth, 35% develop neurological diseases, including hydrocephalus, microcephaly, and mental disability, 80% have eye damage and up to 40% of the children progress to hearing loss (Dubey et al., 2012).

Although hearing loss related to congenital toxoplasmosis has been reported in several studies (Eichenwald, 1960; Wilson et al., 1980; Mcleod et al., 2004; Andrade et al., 2008; Salviz et al., 2013) its pathophysiology remains unclear.

It is presupposed that hearing loss in congenital toxoplasmosis is due to a postnatal inflammatory response. Cysts of *Toxoplasma gondii* were identified in the internal auditory canal, spiral ligament, stria vascularis, and saccular macula of the inner ear. Hearing loss can be secondary to preventable delayed reactivation from the cystic to the active tachyzoite form (Salviz et al., 2013).

Immune response to *Toxoplasma gondii* infection involves three main pathways of the innate immune response, including the Toll-like receptors (TLRs), the IFN-inducible GTPases, and inflammasomes (Zhao and Ewald, 2020). TLR and IL-1 receptor (IL-1R) signaling through Myeloid Differentiation Factor 88 (MyD88) and TNF Receptor-Associated Factor 6 (TRAF6) activate Nuclear Factor kappa B (NF-kB) and Mitogen-Activated Protein Kinase Kinase (MAPK) pathways leading to the production of the interleukins (ILs) 1 (IL-1) and 12 (IL-12; Koblansky et al., 2013), that generate interferon-gamma (IFN-γ) and a Th1-polarized response to *Toxoplasma gondii*. The inflammasome sensors Nod-like receptors (NLRs), NLRP1 (in mice), and NLRP3 (in mice and humans) have been shown to activate proteases that amplify inflammatory signals, like IL-1β release. This process results in caspase-1–mediated processing of IL-1α, IL-1β, and IL-18, triggering apoptosis (Coutermarsh-Ott et al., 2016). Open questions regarding the molecular mechanisms involved in the inflammasome responses to *Toxoplasma gondii* include what parasite signals activate the inflammasome, why proptosis is not engaged, and whether this is the result of active parasite manipulation. Recent data also suggest the existence of a crosstalk between the inflammasome and the IFN-inducible GTPases, a pathway that surveys the cell for foreign or damaged membranes and targets them for clearance downstream of IFN-γ (Lima and Lodoen, 2019; Zhao and Ewald, 2020; Frickel and Hunter, 2021).

The prevalence of sensorineural hearing loss reaches 28% in children that do not receive treatment. Studies suggest that this number can be reduced to zero in early diagnosed patients that receive treatment (Mcleod et al., 2004; Brown et al., 2009).

### 5.2. Rubella

Rubella virus belongs to the family of *Togaviridae*. The single-stranded RNA genome in plus-strand orientation encodes non-structural proteins (e.g., P150 and P90) and structural proteins such as the capsid protein, the glycoprotein E1, and the glycoprotein E2 (Kräter et al., 2018).

In immunocompetent adults, the virus has a self-limited course which is characterized by low fever, pain related to eye movement, conjunctivitis, sore throat, malaise, headache, nausea, decreased appetite, transient arthritis, and sensitive lymphadenopathy (Lee and Scott Bowden, 2000; Frieden et al., 2013). Nevertheless, when acquired during pregnancy, it may be associated with fetal hearing loss, congenital cataracts, microcephaly, intellectual disability, thrombocytopenia, cardiac abnormalities, and cutaneous rash (Pandey et al., 2013).

Sensorineural hearing loss is a complication caused by different viral infections. Viruses can directly damage the structures of the inner ear or activate inflammatory processes that cause hearing loss. The virus-induced hearing loss can be mild, severe to profound, and mainly sensorineural. Sensorineural hearing loss occurs in 58% of the cases of congenital rubella infection. It is more frequent when the maternal rubella infection takes place in the first trimester of pregnancy. The virus causes direct cochlear damage and cell death in the organ of *Corti* and in the *stria vascularis* (Lee and Scott Bowden, 2000). However, it spares vestibular functions (Webster, 1998). Despite rubella’s severe consequences, vaccination is effective against congenital rubella (De Leenheer et al., 2011).

One study indicated that the Rubella virus results in cytoskeleton changes. For instance, the infection of Vero cells by the Rubella virus induces important changes in the filamentous-actin (F-actin) distribution. Moreover, a positive correlation was demonstrated between the cortical (F-actin) and cellular stiffness. Significant changes in cellular stiffness induced by the Rubella virus were characterized by a reduction in both collective and single-cell migration and changes in cell morphology, which in turn was associated with the activation of caspase 3/7, a mark of apoptosis (Kräter et al., 2018). Nonetheless, to which extent these events are related to alterations that the Rubella virus infection promotes in auditory functioning must be further explored.

### 5.3. Cytomegalovirus

The human cytomegalovirus (HCMV) belonging to the *Herpesviridae* family displays a double-stranded DNA genome of 236 kb (Dolan et al., 2004). The translated products encoded by the HCMV genome are much more numerous than previously believed owing to the presence of viral short open reading frames (ORFs), alternative splicing, and translation of cytosolic transcripts outside of conserved reading frames (Herbein, 2018). HCMV is transmitted through direct contact with body fluids such as saliva, tears, urine, stool, semen, and breast milk (Andrei et al., 2009). Congenital infection occurs as a result of either a primary or a recurrent infection acquired during pregnancy (Grosse et al., 2008).

Hearing loss is a common sequela of congenital CMV infection. It affects 10–15% of the infected children and can be either unilateral or bilateral. Hearing loss associated with CMV infection varies from mild to severe. Approximately half of the hearing losses due to congenital CMV infection have a late-onset or are progressive. Therefore, this infection cannot be detected at birth (Fowler and Boppana, 2006; Grosse et al., 2008). Congenital CMV infection is the leading cause of sensorineural hearing loss (SNHL), occurring in 30–65% of children symptomatic at birth and 7–15% of children with asymptomatic infections. In both cases, it is an important cause of permanent bilateral hearing loss (PBHL; Gabrielli et al., 2013). PBHL is classified into conductive, sensorineural, and mixed. Conductive hearing loss originates from impairment in the middle ear that prevents sounds from being efficiently transferred throughout the outer ear canal to the eardrum *via* the ear ossicles. Sensorineural hearing loss is caused by damage in the inner ear or in the auditory nerve and it is permanent. A “mixed” loss means that components of both conductive and sensorineural hearing losses are present (Grosse et al., 2008). Around 50% of the children with SNHL will continue to have further deterioration or progression of their hearing impairment. Another characteristic of CMV-related hearing loss is fluctuating hearing loss, which cannot be explained by concurrent middle ear infections. Fluctuating hearing loss may occur in only one ear or at specific frequencies within the ear. It may compromise both ears leading to PBHL (Fowler and Boppana, 2018).

The neurological changes associated with CMV infections are related to direct virus damage to the cells of the brain parenchyma or to the related inflammatory response (Gabrielli et al., 2009; Enders et al., 2011). Interestingly, corticoid treatment in a mice model of CMV infection was described to decrease the expression of inflammatory mediators such as tumor necrosis factor-alpha (TNF-α) and gamma interferon (IFN-y), thus preserving normal CNS development (Kosmac et al., 2013). Recently, to prove the importance of inflammation in a mice model of CMV, Seleme and colleagues treated infected mice with TNF-α neutralizing antibodies (TNF-NaAbs; Seleme et al., 2017). The authors described that this treatment stabilized the neurodevelopment in the infected mice without affecting the virus replication. Another group showed that inflammation represents an important component of hearing loss related to CMV infection (Bradford et al., 2015). Among this, Almishaal et al. (2020) found that CMV infection reduces wave amplitude in mice and causes a reduction in the number of ribbon synapses per inner hair cell.

The analysis of brains infected congenitally reveals areas of necrosis, containing infiltrating macrophages and microglia, which produce nitric oxide, an important component of the innate immune response that occurs during infection. One study that used a model based on 3-dimensional cortical organoids showed that nitric oxide inhibits human CMV (HCMV) spread and at the same time produces tissue disorganization (Mokry et al., 2022). Moreover, the same study showed that nitric oxide reduces the replication of HCMV in 2-dimensional cultures of neural progenitor cells (NPCs). This is a significant finding, since NPCs are pronounced cells in cortical organoids that differentiate into glial cells and neurons. Therefore, in cases of congenital infection by the HCMV, nitric oxide generates developmental defects that weaken its antiviral activity. Although the specific mechanisms by which HCMV induces sensory changes and more specifically hearing loss has not been yet clarified. The infection of S-phase fibroblast cells by HMCV induces damage at the band position 1q23.3, with a breakpoint located between two loci involved in hearing impairment loci (e.g., DFNA49 and DFNA7) and proximity to the MPZ gene, which is associated with autosomal dominant Charcot–Marie-Tooth syndrome with auditory neuropathy (Nystad et al., 2008).

Prevention of CMV remains elusive. The efficacy of vaccination or passive immunization with hyperimmune globulin has been studied in clinical trials. However, they are still not offered for clinical use. Thus, so far there are no vaccines available to prevent or limit CMV infection in pregnant women. In this scenario, behavioral and educational interventions are the most effective strategies to prevent maternal CMV infection (Goderis et al., 2014; Fowler and Boppana, 2018).

### 5.4. Human immunodeficiency virus

Acquired immune deficiency syndrome (AIDS) was recognized as a disease in 1981. It is caused by the human immunodeficiency virus (HIV), previously known as lymphadenopathy-associated virus (LAV) or human T-lymphotropic virus type III (HTLV-III; De Clercq, 2009). HIV infects cells of the immune system, destroying them and impairing their function. Therefore, HIV infection results in a progressive deterioration of the immune system. The immune system is considered deficient when it can no longer fulfill its role of responding to infection and disease. Infections associated with severe immunodeficiency are known as opportunistic (WHO, 2017).

HIV infects approximately 35 million people worldwide. Over the past decade, the quality of healthcare assistance, as well as the access to antiretroviral therapy (ART), have improved worldwide. These measures have reduced HIV-related morbidity and mortality (Hrapcak et al., 2016). HIV infection affects multiple organs and tissues, including the inner ear structures, the central auditory pathways, and the cortical areas associated with auditory processing. When such structures are affected, HIV may produce hearing impairment (Maro et al., 2016).

In some cases, the CNS may even serve as a reservoir of HIV (Mirza and Rathore, 2012). The involvement of the CNS, including the structures of the auditory pathway, is considered as the leading cause of the auditory abnormalities connected to HIV infection (Christensen et al., 1998). The hearing deficits are either sensorineural, conductive, or both. Conductive hearing loss occurs when the conduction of the sound is obstructed through the external ear, the middle ear, or both. On the other hand, sensorineural hearing loss occurs when there is some structural or functional change inside the cochlea or in neural pathway to the auditory cortex (Maro et al., 2016). The hearing impairment related to HIV infection and AIDS may be a direct consequence of the viral infection or related to the pharmacologic treatment used to treat the disease. In children, generalized lymphadenopathy, oropharyngeal candidiasis, and parotid hypertrophy are the most prevalent otolaryngologic manifestations of HIV infection (Sturt et al., 2014). Nonetheless, so far, few studies have investigated the epidemiological aspects as well as the mechanisms underlying the hearing loss driven by HIV infection in children. Significant discrepancies in the prevalence of hearing loss in HIV-infected children are found among the available studies, ranging from 6.4 to 84.8%, which could be a reflex of differences in the studied populations (Weber et al., 2006; Torre et al., 2012; Hrapcak et al., 2016; Ianacone et al., 2017). Hearing loss in HIV may arise from recurrent otitis media as well as from opportunistic infections such as CMV infection, tuberculosis, cryptococcosis, syphilis, and bacterial meningitis (Hrapcak et al., 2016). It has been suggested that some of the retroviral medications used to treat HIV, such as certain nucleoside reverse transcriptase inhibitors (NRTIs), may lead to sensorineural hearing loss (Kakuda, 2000). However, the results of a prospective study conducted with the NRTIs zidovudine and didanosine do not support this hypothesis (Schouten et al., 2006).

There is an urgent need for improved screening tools, identification methods, and treatment of hearing problems in HIV-infected children. Screening strategies need to be developed and tested since caregivers are not proficient in identifying hearing loss (Hrapcak et al., 2016; Ensink and Kuper, 2017). It has been suggested that the hearing loss related to HIV infection is related to central rather than peripheral mechanisms (Buckey et al., 2019). In fact, a clinical study that evaluated the ABR in neonates showed that 11.1% of HIV-exposed and 6.6% of unexposed newborns presented hearing impairment (*p* = 0.2214), and that the hearing thresholds of HIV-exposed newborns was correlated with maternal viral load (*p* = 0.034), while the maternal CD4 cell counts was not (*p* = 0.059; Fasunla et al., 2014). These findings support the existence of changes in the auditory pathway with a trend of more hearing loss in HIV-exposed newborns and indicates the need of more studies that explore the relationship between newborn hearing loss and *in-utero* exposure to HIV.

### 5.5. Syphilis

Syphilis is a sexually transmitted disease caused by the bacterium *Treponema pallidum*. Nonetheless, little is known in relation to its mechanism of action or what determines the aggressiveness of syphilis infection (De Santis et al., 2012). In 2017, the incidence of pregnant women diagnosed with syphilis was higher than 1% in 37 out of 83 countries whose data were available (WHO, 2017). Congenital syphilis may cause periostitis, facial and skin deformities, hepatosplenomegaly, intellectual disability, skin rash, hydrocephalus, lymphadenopathy, meningitis, and sensorineural hearing loss (Bale and Murph, 1992; Ressler and Nelson, 2000).

The clinical sequelae of *Treponema pallidum* infection are divided into three stages. Primary syphilis is characterized by the presence of a “chancre” at the site of treponemal inoculation. Secondary syphilis represents the phase of hematogenous dissemination, which is commonly followed by a latent or asymptomatic period. Following this latent phase, patients may progress to develop tertiary syphilis, which occurs years or even decades after the infection (Pletcher and Cheung, 2003). Tertiary syphilis can affect the nervous system (neurosyphilis), the cardiovascular system (cardiovascular syphilis), or the skin. This may occur in roughly 40% of the unmedicated patients (WHO, 2017).

Unfortunately, a high prevalence of congenital syphilis is still reported in many countries (WHO, 2017). The transmission of syphilis during pregnancy mostly occurs *via* a transplacental route tough transmission during birth is also possible (Stoltey and Cohen, 2015).

In infants, hearing loss related to congenital syphilis has been well-documented (Nadol, 1975; Chau et al., 2009). However, very little progress has been made to understand the pathophysiology involving fetal infection as well as how and where the *Treponema pallidum* affects ear development (Becker, 1979; Peeling and Hook, 2006).

Healthcare programs for hearing screening of newborns have been initiated in many countries in an effort to identify the hearing loss related to congenital syphilis at an early age (American Academy of Pediatrics, 2007). This is a crucial measure since exposure to syphilis in newborns is considered a risk to the development of sensorineural hearing loss (Chau et al., 2009).

The hearing loss related to late congenital syphilis during childhood is sudden, bilateral, and symmetric (Chau et al., 2009). The hearing loss of otosyphilis typically begins at a high frequency, progressing to a bilateral and complete loss of the cochlear and vestibular functions. Congenital syphilis has a faster course, with a pattern of symmetric hearing loss and less tinnitus/vertigo than the one found in adults. In patients with concurrent HIV infection, the time course of otosyphilis is accelerated (Pletcher and Cheung, 2003).

Otosyphilis has been defined as the presence of a positive rapid plasma regain (RPR) in the presence of unexplained sensorineural hearing loss (Becker, 1979). The classic symptoms include unexplained hearing loss and Meniere’s syndrome, with fluctuating tinnitus and episodic vertigo. Several pathophysiological mechanisms, including direct perilymphatic invasion by spirochetes, temporal bone osteitis, and microvascular inflammation have been proposed (Pletcher and Cheung, 2003).

Syphilis is very sensitive to penicillin, consequently, benzathine penicillin is the treatment of choice. *Treponema pallidum* has a long incubation period. Therefore, contact investigation and prophylactic treatment of asymptomatic exposed contacts can abort the ongoing spread of the infection. Treatment of the infected mother during pregnancy significantly reduces the chance of congenital syphilis. Therefore, the identification and treatment of pregnant women diagnosed with syphilis is a public health priority (Stoltey and Cohen, 2015).

As described by Nadol (1975), the inner ear impairment caused by syphilis in congenital infantile starts as a meningo-neuro-labyrinthitis, which is characterized by round cell infiltration of the labyrinth and vestibulocochlear nerve (Nadol, 1975). This characterization complemented Goodhill’s work that described an extreme degeneration of the organ Corti and of the spiral ganglion as well as labyrinth hemorrhage and fibrinous accumulation. Moreover, Nadol’s findings corroborate Goodhill’s report, by observing round cell infiltration of nerve fibers (Goodhill, 1939). A further study showed that different types of cells such as macrophages, lymphocytes, and plasma cells compose this infiltrate (Engelkens et al., 1993). Another study described the presence of inflammatory mediators’ transcripts such as IFN-γ, TNF-α, C-C motif chemokine ligand 2 (CCL2), C-X-C motif chemokine (CXCL10) in patients’ lesions (Cruz et al., 2012). In addition, increased expressions of the cytokines IL-2 and IFN- γ were found in syphilis lesions (Van Voorhis et al., 1996; Podwinska et al., 2000; Salazar et al., 2002; Stary et al., 2010). Altogether, this evidence suggests a role for cell migration in the inflammatory modulation of inner ear damage.

### 5.6. Sars-CoV-2

The betacoronaviruses genus comprises the SARS-CoV viruses (severe acute respiratory syndrome coronavirus), MERS-CoV (Middle East respiratory syndrome coronavirus) and the SARS-CoV-2 (severe acute respiratory syndrome 2 coronavirus; Almeida and Tyrrell, 1967; Kapikian et al., 1969.; Peiris et al., 2004; Woo et al., 2005; Zaki et al., 2012). The SARS-CoV-2 is responsible for the COVID-19 (Zhu et al., 2020), which was declared a pandemic by the World Health Organization (WHO) on March 11, 2020.

Fever, cough, fatigue, and gastrointestinal changes are among the main clinical symptoms of COVID-19 (Guo et al., 2020). The infection by the SARS-CoV-2 should be closely monitored in elderly patients as well as in patients with comorbidities, pregnant women, and newborns.

Being in the third trimester and having comorbidities puts pregnant women at particular risk when diagnosed with COVID-19 (Turan et al., 2020). Pregnant women may progress to severe pneumonia when infected by the SARS-CoV-2 due to physiological and immunological changes that take place during the perinatal period, such as changes in T lymphocyte immunity, increased oxygen consumption, decreased functional residual capacity, and decreased chest compliance which results in increased maternal and fetal morbidity and mortality (Tang et al., 2018).

SARS-CoV-2 may cause direct destruction of inner ear structures, especially the hair cells of the inner ear. It has been suggested that SARS-CoV-2 may act by either destroying the organ of Corti or activating the host immune system (Mustafa, 2020).

Otoacoustic emissions (OAE) are characterized by acoustic signals resulting from the activity of OHC of the inner ear and are used to measure lesions in OHC, displaying high sensitivity to cochlear insults. Suppression of OAE is an essential clinical tool to detect the inhibitory efferent role of the central auditory system in cochlear processes, which perform a critical task in speech perception (Celik et al., 2021). Celik et al., assessed the cochlear functions of 37 toddlers that had been exposed to intrauterine SARS-CoV-2 and observed low amplitudes of transient evoked otoacoustic emission (TEOAE) in patients at high frequencies (3–4 kHz) and poor contralateral suppression activity, especially at higher frequencies (2.3.4 kHz). The authors suggested that such observations resulted from a deficiency in the efferent medial olivocochlear system in toddlers exposed to intrauterine SARS-CoV-2 (Celik et al., 2021). Similar studies have demonstrated that regardless of age, SARS-CoV-2 may cause hearing impairment in patients with asymptomatic COVID-19 (Kilic et al., 2020; Mustafa, 2020; Alves de Sousa et al., 2021).

Although the mechanisms of brain injury in COVID-19 are still poorly understood, other members of the coronavirus family have already been associated with neurological diseases (Huang et al., 2020). SARS-CoV-2 binds to the angiotensin-converting enzyme receptor 2 (ACE2) to access human cells (Lu et al., 2020). The middle ear has a relatively high expression of ACE2 and there is evidence that SARS-CoV-2 colonizes the epithelium of the middle ear (Kurabi et al., 2022). Inner ear cells co-express all the machinery necessary for SARS-CoV-2 infection and replication, which includes not only ACE2 but also the transmembrane protease serine 2 (TMPRSS2) and furin (Jeong et al., 2021). Schwann cells and hair cells explanted from the human vestibular organ are permissive to SARS-CoV-2 infection. Moreover, otic prosensory cells (OPCs) and Schwann cell precursors (SCPs), which are two-dimensional models of human induced pluripotent stem cell (hiPSC)-derived *in vitro*, express ACE2, TMPRSS2, and FURIN. SCPs also permit SARS-CoV-2 infection (Jeong et al., 2021). Three-dimensional ear organoids generated for ear infection also showed that hair cells express ACE2 and are targets for SARS-CoV-2 (Jeong et al., 2021).

Central glial cells and neurons, in addition to endothelial cells and arterial smooth muscle cells of brain arteries, express ACE2, which makes them potential targets of the SARS-CoV-2 (Alenina and Bader, 2019; Baig et al., 2020; Zou et al., 2020). Chen and colleagues demonstrated that most ACE2 are found in both neuronal and non-neuronal cells of the human middle temporal gyrus and the posterior cingulate cortex (Zou et al., 2020). They also showed that glial cells, mainly astrocytes and oligodendrocytes, are positive for ACE2 in all areas of the human brain, while the microglia are only positive in the human middle temporal gyrus. The authors also found high expression of ACE2 in the olfactory bulb and in endothelial cells, raising the hypothesis that the access of SARS-CoV-2 to the brain would occur through the olfactory bulb, thus bypassing the blood–brain barrier (BBB; Chen et al., 2021).

The so-called cytokine storm is an important life-threatening feature of the SARS-CoV-2 infection (Coperchini et al., 2020; Huang et al., 2020; Mehta et al., 2020). This phenomenon is characterized by the production of high levels of inflammatory mediators (e.g., interferons, TNFα, interleukin 1 beta - IL-1β, IL-6, and IL-10), chemokines, and colony-stimulating factors (CSFs; Tisoncik et al., 2012; Coperchini et al., 2020), which are increased in patients with severe forms of COVID-19 (Henry et al., 2020; Huang et al., 2020; McGonagle et al., 2020). The cytokine storm could also be responsible for some of the neurological symptoms of COVID-19, by promoting the permeability in the BBB and neuronal damage regardless of direct viral infection.

The analysis of severe and moderate cases of COVID-19 demonstrated an increase in CNS injury biomarkers such as the glial fibrillar acidic protein (GFAP) and neurofilament light (NfL) chain protein, suggesting activation of astrocytes and neuronal injury in such patients (Kanberg et al., 2020). Although neurotropism has been shown for other coronaviruses, the infection of glial cells by SARS-CoV-2 still needs stronger evidence, especially from *in vivo* studies (Vargas et al., 2020).

### 5.7. Zika virus

Zika virus (ZIKV) is a single-stranded RNA virus that belongs to the *Flaviviridae* family and to the genus flavivirus. Viruses of more than 70 species are inserted into serologically associated groups. These include yellow fever virus (YFV), dengue virus (DENV), Japanese encephalitis virus (JEV), and West Nile virus (WNV; Lanciotti et al., 2008). ZIKV RNA encodes three structural proteins and seven non-structural proteins. Phylogenetic analyzes identified two main strains of ZIKV, termed African and Asian (Lanciotti et al., 2008; Haddow et al., 2012).

Zika is an acute viral disease caused by ZIKV, mainly transmitted by a mosquito of the *Aedes* genus. This comprises the *Aedes aegypti* and the *Aedes albopictus* (Dick and Kitchen, 1952; Smithburn, 1952; Plourde and Bloch, 2016; Yee et al., 2020). This disease is characterized by pruritic maculopapular rash, intermittent fever, non-purulent conjunctival hyperemia without pruritus, arthralgia, myalgia, and headache. These features are comparable to the symptoms found in infections caused by other arboviruses. Hence, the clinical diagnosis must be puzzling. Most cases follow a benign course, and the symptoms usually disappear spontaneously within a period of 2–7 days. Around 80% of the people infected by ZIKV are asymptomatic (Duffy et al., 2009). However, deaths due to the disease have been reported. Moreover, a surge of microcephaly and neurological manifestations related to congenital Zika was widely reported during the epidemic of ZIKV infection that affected South and Central America as well as the Caribbean, in 2015 (Mlakar et al., 2016). Such clinical manifestations find support in experiments performed in mice that demonstrated the neurotropism of the ZIKV (Dick and Kitchen, 1952).

Human cases of ZIKV infection, confirmed by blood tests, were reported in Asia and Africa in the 1960s and 1980s. Prior to 2007, only 14 cases were reported, during the first major outbreak on Yap Island (Federated States of Micronesia). Those were the first cases reported out of Asia and Africa (Duffy et al., 2009). In February 2014, cases of ZIKV infection were reported in the Americas for the first time (Fauci and Morens, 2016). In March 2015, the virus was first registered in South America, when cases of ZIKV infection were registered in the northeast states of Brazil (Campos et al., 2015). After that, the association between ZIKV infection and neurological disorders such as Guillain-Barré syndrome was reported (WHO, 2016a,b,c).

In October 2015, an unusual rise in the number of cases of congenital microcephaly was reported in Brazil. This was declared a national public health emergency in the following months, amid a sustained increase in the number of cases of microcephaly (De Albuquerque et al., 2016). In September 2016, a study concluded that ZIKV infection in pregnant women could cause congenital brain abnormalities including microcephaly (WHO, 2016a,b,c). It has been estimated that up to 20 to 30% of the infected pregnant women can transmit ZIKV to the fetus, of which 4 to 7% progress to abortions, while 5 to 14% give rise to fetuses and neonates with congenital zika syndrome (CZS; Musso et al., 2019). CZS refers to a spectrum of birth defects that comprises several types of CNS disorders (Musso et al., 2019).

CZS is represented by microencephaly, ventriculomegaly, brain calcifications, brainstem defects, sensory system alterations (e.g., chorioretinal atrophy, lens subluxation, hearing loss), and muscle contractures (De Oliveira Melo et al., 2016; Ventura et al., 2016; Moore et al., 2017). In a work performed in Brazil, 66% of the studied children exhibited ventriculomegaly and 14 children had cerebral atrophy (De Albuquerque et al., 2016). The eyes are also among the main targets of the teratogenic action of the ZIKV (Mlakar et al., 2016).

According to the Brazilian epidemiological bulletins, 19.622 suspected cases of CZS were reported, of which 3.577 (18.2%) were confirmed by exams, from 2015 to 2020. Only in 2020, 1.007 new cases were reported, and 35 of those (3.5%) were confirmed [MS (Ministério da Saúde), 2021]. Therefore, even though the emergency period has ended, new cases of CZS continued to occur in Brazil.

The brain is an organ by which ZIKV has a tropism and is one of the most affected in transplacental infections (Garcez et al., 2016; Zare Mehrjardi et al., 2017; De Paula Guimarães et al., 2019; Teixeira et al., 2020). Two hypotheses regarding the role of the placenta in CZS have emerged. The placenta could directly transmit the ZIKV to the embryo/fetus at an early stage or the placenta itself could create a response to the ZIKV exposure; this response could contribute to or even be the cause of brain defects (Adibi et al., 2016).

ZIKV has an affinity for neural tissue. Electron microscopy findings suggest possible viral persistence in the fetal brain, possibly due to the immunologically safe environment for the virus (Rasmussen et al., 2016). Viral tropism for neuronal cells occurs at any stage of neurodevelopment. ZIKV has been isolated from brain tissue and cerebrospinal fluid (CSF) of children with congenital syndrome (Costello et al., 2016). *In vitro* findings have also demonstrated that ZIKV can interfere with all stages of neurogenesis (Vouga and Baud, 2016). The alterations caused by ZIKV in the cells of the nervous system are not restricted only to neurons but can also affect glial cells, as was recently discussed in the literature (Quincozes-Santos et al., 2023).

Some studies have explored the entry of ZIKV in different cell types. The Axl receptor, part of the TAM tyrosine kinase family, appears to be one of the most important for ZIKV entry into different cell types. Both *in vitro* and *in vivo* studies have confirmed that human neural progenitor cells, which are permissible for ZIKV infection, express Axl (Tang et al., 2016). Axl mRNA is also present in other brain cells, including radial glial cells, astrocytes, and microglial cells (Nowakowski et al., 2016; Meertens et al., 2017; Rothan et al., 2019).

*In vitro* studies conducted by Bayless and colleagues have shown that ZIKV infects cranial neural crest cells (CNCCs). CNCCs give rise to most cranial bones and have paracrine effects on the developing brain. Infected CNCCs experience limited apoptosis. However, they secrete cytokines that promote cell death and lead to aberrant differentiation of neural progenitor cultures. The addition of cytokines (LIF - leukemia inhibitory factor or VEGF - vascular endothelial growth factor) in quantities similar to those secreted by CNCCs infected with ZIKV is sufficient to recapitulate early neuronal differentiation and apoptotic death of neural progenitors, contributing to embryopathies (Bayless et al., 2016).

The results of a study conducted by Garcez et al. revealed that ZIKV induces cell death, with a similar time frame and magnitude, in both iPS (induced pluripotent stem cells)-derived neural stem cells and in radial glia-like cells (Garcez et al., 2017). These results suggest that the same targets are likely affected by ZIKV in both cell types. Interestingly, glial cells, and not neurons appear to be the main targets of ZIKV, suggesting that cells with proliferative capacity are preferred (Retallack et al., 2016; Tang et al., 2016; Garcez et al., 2017).

Cultures of myelinating cells from the CNS and from the peripheral nervous system (PNS) of wild-type mice and mice knockout to Ifnar1 (interferon alpha and beta receptor subunit 1) were infected by ZIKV to examine the neuronal and glial tropism of ZIKV and the short-term consequences of a direct infection with the Brazilian variant of ZIKV (Cumberworth et al., 2017). According to the results of this study, CNS cells are considerably more susceptible to ZIKV infection than PNS cells. The authors demonstrated that axons of CNS neurons and myelinating oligodendrocytes are particularly vulnerable to the injuries caused by ZIKV infection. In addition, ZIKV infection is more prominent when type I interferon responses do not occur (Cumberworth et al., 2017).

The brain damage related to ZIKV includes microcephaly, lissencephaly, cerebellar hypoplasia, and ventriculomegaly (Martines et al., 2016). Noteworthy, the association between microcephaly and other neurological injuries is so relevant that the finding of isolated microcephaly should not be considered a marker of congenital ZIKV infection (Carvalho et al., 2016; De Oliveira-Szejnfeld et al., 2016; Vouga and Baud, 2016; Schwartz, 2017).

Garcez et al. examined the effects of ZIKV infection on human neural stem cells growing as neurospheres and brain organoids. The authors showed that ZIKV reduces the viability and growth of both neurospheres and brain organoids. These results suggest that ZIKV impairs neurogenesis during human brain development (Garcez et al., 2016).

Cell death can be the cause of the reduction in the size of the brain, resulting in microcephaly. However, other mechanisms might also occur concurrently with cell death in ZIKV infection. In fact, it has been demonstrated that delays in the cell cycle produce a reduction in neuronal generation (Lancaster et al., 2013), and may be responsible for the reduction in brain size. Garcez et al. provided insights into the molecular mechanisms behind ZIKV self-replication, the associated disruption of the cell cycle, the negative regulation of neurogenesis, and the increased cell death (Garcez et al., 2017).

Other neurological findings of CZS are hydrocephalus, diffuse astrocytic, cerebellar dysgenesis, abnormal development of the *corpus callosum*, irregular groove areas, polymicrogyria, lissencephaly and abnormalities in the cortical migration (De Oliveira Melo et al., 2016; Mlakar et al., 2016; Vouga and Baud, 2016; Schwartz, 2017).

Cell death of nerve fibers is an additional finding that has been reported (Martines et al., 2016; Azevedo et al., 2018). However, other pathological findings such as edema, vascular congestion, calcifications, and lymphocytic infiltrate have already been described in the brain tissues of fatal cases (Azevedo et al., 2018; Aragão et al., 2019). The development of fetal brain lesions following the inoculation with ZIKV in a pregnant monkey was marked by periventricular lesions that developed within a period of 10 days and evolved asymmetrically to the occipital-parietal lobes (Adams Waldorf et al., 2016).

Little is known regarding the prevalence of hearing loss in cases of CZS (Leal et al., 2016a,b). Furthermore, it is unclear if a possible significant hearing loss related to ZIKV infection during pregnancy would be associated with the effects of the virus in the outer or middle ear (conductive hearing loss), in the inner ear or even in the CNS (sensorineural hearing loss). Evidence of a presumed sensorineural hearing loss was detected in 6–9% of the microcephalic cases from the Brazilian outbreak (De Albuquerque et al., 2016; Leal et al., 2016a,b). Reported reductions in the OAE imply the occurrence of a decreased electromotility of the inner ear’s sensory cells in response to sound, while decreased brainstem auditory responses may result in damage somewhere throughout the cochlear auditory pathway to the midbrain. It has been shown that the changes in the OAE range from 0 to 75%, while the changes in the brainstem auditory evoked potential range from 0 to 29.2% (de Barbosa et al., 2019; Fadiño-Cárdenas et al., 2019).

The incidence of ZIKV-associated hearing loss, although transient, has also been reported in mice, where 25–66% of the animals born to immunocompromised mothers that were infected during pregnancy showed decreased brainstem auditory responses, depending on the stimulus frequency (Julander et al., 2018).

The changes associated with ZIKV infection that take place in sensory organs probably derive from the neurotropic nature of the virus, as demonstrated by studies with neuronal derivatives of human stem cells, non-human primates’ cells, and cells from mice. These studies revealed that ZIKV proliferates more in neural progenitors than in mature neurons. In addition, infected cell populations show reduced proliferation and increased cell death (Frieden et al., 2013; Garcez et al., 2016; Miner and Diamond, 2017; Qian et al., 2017). Similar results have been reported in studies performed on chicken embryos (Goodfellow et al., 2016; Thawani et al., 2020).

The sensory organs derived from the placode contain neural-like receptor cells, and the cranial placodes also give rise to cranial ganglion neurons. The otic placode gives rise to sensory organs and the statoacoustic ganglion; these structures are potential targets of the ZIKV (Thawani et al., 2020).

The morphogenesis of the human inner ear structures takes place within the first 10 weeks of gestation and the labyrinth reaches its mature size by the 19th week (Lim and Brichta, 2016). Interestingly, during this timeframe, most mothers of newborns with hearing loss associated with ZIKV reported a skin rash and/or other symptoms of infection (Leal et al., 2016b). Therefore, it is likely that there is a temporal window of viral susceptibility of the inner ear epithelium. Direct injury to the auditory organ by the ZIKV or local inflammatory changes induced by a ZIKV infection are probably the mechanisms involved in the hearing changes linked to ZIKV (Barbosa et al., 2019).

ZIKV-induced inflammation has been reported by several authors (Hamel et al., 2015; Shao et al., 2016; Lum et al., 2017; Diop et al., 2018). The effects of interleukin IL-1β and TNF-α, causing injuries to vasculature and neurons, suggest a role of these two pro-inflammatory cytokines in the upregulation induced by ZIKV in human skin fibroblasts (Friedl et al., 2002; Lu et al., 2005; Puhlmann et al., 2005; Hamel et al., 2015). Moreover, the microglia cell line CHME5 infected with ZIKV caused an increase in the expression of IL-6, TNF-α, IL-1β, inducible nitric oxide synthase (iNOS), and nitric oxide (NO), corroborating the role of neuroinflammation in the disease (Diop et al., 2018). Such results strongly suggest that an exacerbated inflammation increase neuronal cell death, thus leading to neurological abnormalities that could be involved in the poor infants’ outcomes. Although the relation between inflammation mediated by ZIKV and congenital hearing loss is still far from being elucidated, this is a fertile field to be explored.

It is known that the early diagnosis and intervention in patients with congenital hearing loss increase the chances of mitigating the prognosis language and communication development impairments. This becomes even more evident in the context of the association of ZIKV with other malformations. Thus, it is imperative to better elucidate the pathogenesis of the involvement of the auditory system in congenital and acquired ZIKV infections to optimize the strategies of monitoring, preventing and treatment used to counteract ZIKV infection (Barbosa et al., 2019).

## 6. Conclusion

This comprehensive review has focused on the hearing loss associated with different types of neonate infection diseases. The epidemiology, as well as the anatomical and pathophysiological basis related to the hearing changes that take place in each of these diseases have been examined. When arising during pregnancy, viral, protozoan, and bacterial infections discussed in this paper such as toxoplasmosis, rubella, CMV, HIV, syphilis, COVID-19, and ZIKA can all compromise maternal and fetal health, and generate cochlear damage that can progress to sensorineural deafness. However, studies that evaluate the relationship between vertical infection and the development of hearing loss are still scarce. Maternal and fetal health are always causes of concern, especially during epidemic periods of these diseases.

## Author contributions

DC and VM-N conceived the project. The manuscript was drafted by MD, LA, PNu, MH, AX, LP, CS, JC-A, CM, LR, and SD. AF, DC, and MD led the writing. MD, PNi, and VM-N revised the manuscript. All authors contributed to the article and approved the submitted version.

## Funding

We thank the Brazilian Government Agencies: Conselho Nacional de Desenvolvimento Científico e Tecnológico (CNPQ); Coordenação de Aperfeiçoamento de Pessoal de Nível Superior (CAPES) and Fundação de Amparo à Pesquisa do Estado do Rio de Janeiro (FAPERJ), for their financial support.

## Conflict of interest

The authors declare that the research was conducted in the absence of any commercial or financial relationships that could be construed as a potential conflict of interest.

## Publisher’s note

All claims expressed in this article are solely those of the authors and do not necessarily represent those of their affiliated organizations, or those of the publisher, the editors and the reviewers. Any product that may be evaluated in this article, or claim that may be made by its manufacturer, is not guaranteed or endorsed by the publisher.
